# Excessive Urinary p75ecd is a Potential Indicator of Amyotrophic Lateral Sclerosis: An American Cohort Study

**DOI:** 10.2174/011570159X352364250212035802

**Published:** 2025-05-09

**Authors:** Swati Dhasmana, Anupam Dhasmana, Sheema Khan, Acharan S. Narula, Shafiul Haque, Murali M. Yallapu, Subhash C. Chauhan

**Affiliations:** 1 Department of Immunology and Microbiology, Medicine and Oncology ISU, School of Medicine, University of Texas Rio Grande Valley, McAllen, TX 78504, USA;; 2 South Texas Center of Excellence in Cancer Research, Medicine and Oncology ISU, School of Medicine, University of Texas Rio Grande Valley, McAllen, TX 78504, USA;; 3 Himalayan School of Biosciences, Swami Rama Himalayan University, Dehradun, India;; 4 Narula Research LLC, 107 Boulder Bluff, Chapel Hill, North Carolina, NC, 27516, USA;; 5 Research and Scientific Studies Unit, College of Nursing and Allied Health Sciences, Jazan University, Jazan, Saudi Arabia

**Keywords:** Amyotrophic lateral sclerosis, p75ecd, biomarker, urine, ELISA, early diagnosis

## Abstract

**Introduction:**

Amyotrophic lateral sclerosis (ALS) is an idiopathic, fatal, and rapidly progressive neurodegenerative disease. At present, neurofilament light (NFL) and phosphorylated neurofilament heavy (pNfH) proteins in biological fluids are commonly known prognostic biomarkers, but their levels stabilize over time. Thus, there is a critical gap in the field to identify unique biomarkers that can aid disease diagnosis, progression and monitoring the therapy response.

**Aims:**

To evaluate the presence of extracellular domain of p75 (p75ecd) in urine of ALS patients and healthy control volunteers in the North American cohort.

**Methods:**

An enzyme-linked immunoassay (ELISA) and creatinine assay was used to determine the levels of p75ecd and creatinine in the urine of ALS patients and healthy control volunteers respectively. This assay demonstrated clear discrimination in the levels of the p75ecd in the urine samples of ALS patients as compared to healthy individuals.

**Results:**

It was found that the concentration of p75ecd in ALS samples was significantly higher than that of healthy controls group. Additionally, high p75ecd levels were segregated with respect to age, sex, family history, occupation and drug treatment, medication status. Moreover, we observed differential expression patterns among the different stages of the disease. Our results followed the pattern that was observed in the Chinese, and Australian cohort.

**Conclusion:**

Altogether, our results indicate that the development of an efficient system for the detection of elevated levels of p75ecd in the urine could serve as a useful modality for early ALS diagnosis, disease progression, and monitoring the effectiveness of therapeutic interventions.

## 1 INTRODUCTION

Amyotrophic lateral sclerosis (ALS) displays as a progressive neurodegenerative disorder demonstrated by the gradual onset of paralysis, predominantly affecting limbs and respiratory muscles. Progressing rapidly, ALS currently lacks a definitive cure, with patients facing a median survival rate of only 36 months, primarily succumbing to complications arising from respiratory muscle paralysis. Therefore, conducting longitudinal studies on ALS patients is challenging. Moreover, ALS presents insidiously, posing difficulties in early detection and identification [1]. It is widely recognized that ALS stems from a multifaceted interplay of environmental and genetic factors, encompassing various pathophysiological mechanisms including oxidative stress, glutamate excitotoxicity, axonal transport disruptions, and mitochondrial dysfunction [2-4]. At present, riluzole and edaravone are the only therapeutic options for slowing the progression of ALS, however, their efficacy remains remarkably constrained. Given the average life expectancy of only 2-5 years post-diagnosis, there exists a prerequisite for diagnostic, prognostic, susceptibility/risk, and therapy response biomarkers. This will be useful to expedite ALS prediction, diagnosis, enhance prognostic predictions, refine clinical trial design, and facilitate the interpretation of trial outcomes.

There is an unmet clinical need for biomarkers development in ALS diagnosis and prognosis. In the last 20 years, numerous biomarker candidates have emerged for ALS [5]. Among these biomarker candidates, neurofilament light (NfL) and phosphorylated neurofilament heavy (pNfH) are considered promising prognostic markers. However, neurofilament levels tend to remain relatively stable over time, thus, failing to reflect disease progression [6]. This has prompted us to consider the neurotrophin receptor (p75) as a potential biomarker for motor neuron degeneration. Some studies suggest that the extracellular domain of p75 (p75ecd) is released in the urine of ALS patients [7-9].

p75 neurotrophin receptor (p75NTR or NGFR) is a single transmembrane protein comprising 399 amino acids. It has an amino-terminal negatively charged extracellular domain (ecd), containing four highly glycosylated tandem cysteine-rich domains (CRD1 to CRD4). Each of these domains span 40 amino acids and contains six cysteine residues crucial for ligand binding. Additionally, the receptor includes a carboxy-terminal intracellular domain (icd) consisting of 155 amino acids. This icd consists of a juxtamembrane (chopper) domain and a death domain [10-12]. The p75NTR receptor primarily expresses in the developing brain, where it plays a major role in axonal pruning mediated by neurotrophins. Furthermore, Schwann cells within the peripheral nervous system express this receptor, which modulates myelination by serving as a coreceptor for myelin-associated glycoprotein binding to the neurite outgrowth protein receptor. p75NTR is weakly expressed in sympathetic and sensory neurons, as well as in specific subsets of enteric and parasympathetic neurons [8]. As a member of the tumor necrosis factor (TNF) receptor superfamily, p75NTR serves as a crucial receptor primarily recognized for its function as a low-affinity pan-neurotrophin receptor. p75NTR associates with the Trk family, Nogo, and sortilin receptors, and transduces signals from nerve growth factor (NGF), brain-derived neurotrophic factor (BDNF), NT3, and NT4/5. These signals regulate a wide range of processes necessary for the development and maintenance of the nervous system [13] p75NTR is expressed in the spinal motor neurons during the embryonic phase but gradually decreases postnatally. While TrkA and p75NTR are not typically expressed in adult motor neurons, p75NTR expression re-emerges following neuronal injury and motor neuron degeneration. The upregulation of p75NTR during neuronal injury contributes to growth cone collapse, axon growth inhibition, and apoptosis of injured neurons. Notably, the extracellular domain of p75NTR (p75ecd) undergoes cleavage upon binding of pro-apoptotic ligands and can be detected in human urine across various age groups. Research indicates that p75ecd is shed and excreted in urine following nerve injury [14]. Extracellular and Intracellular domains of p75NTR are cleaved enzymatically by an α-secretase (ADAM17) and γ-secretase, respectively. The increased enzymatic activity of secretases leading to cleavage of p75NTR is linked to the sciatic neurons demise, after injury [15]. Both intracellular and extracellular domains are associated with death signaling of the motor neurons [16]. Studies also suggest the presence of p75ecd in the urine of ALS patients [6, 7, 9, 14, 17, 18].

Herein, we have determined the levels of p75ecd in the urine samples of ALS patients using a North American cohort. Demographic studies are crucial in biomarker studies as they provide a universal understanding of the assay and provide information if demographic factors that might influence the biomarker levels. Additionally, combining biomarker information with demographic information might help develop a more accurate and precise test with biological variations [19]. Our study demonstrated the association of p75ecd levels with ALS, type of ALS, age, family history, occupation, and status of medication (Rilutek). These results also suggest that p75ecd could also be a useful biomarker of ALS for the North American population.

## 2 MATERIALS AND METHODS

### 2.1 Participants, Samples, and Data Collection

Two groups of participants, the ALS group and the normal control group were included in the study, with matching gender and age. All the urine samples for the ALS group were procured from the National ALS Biorepository (U.S. Centers for Disease Control and Prevention, Atlanta, Georgia USA). The samples were non-identifiable and had unique codes provided by the NALS biorepository. All the urine samples for normal control were from healthy volunteers. All the samples were procured/collected according to the University’s Institutional Biosafety Committee (IBC) guidelines. This study was evaluated and approved by the Institutional Review Board (IRB). The ethical approval number for this study is IRB-21-0207.

### 2.2 Measurement of p75ecd in Urine by ELISA

Each sample was quantified in triplicate using a sandwich ELISA. The levels of p75ecd were evaluated using an ELISA kit by R&D systems (Cat No: DY367) using the manufacturer’s protocol. Similarly, the same samples in triplicate were also evaluated for the levels of creatinine. Creatinine was quantified using a colorimetric detection kit by Enzo (Cat No: ADI-907-030A) following the manufacturer’s protocol. The urinary p75ecd concentrations were normalized by creatinine concentration in the same sample and the final levels of p75ecd were calculated in ng/mg of creatinine.

### 2.3 Statistical Analysis

GraphPad Prism 5 (GraphPad Software, Inc., La Jolla, CA, USA) was used to analyze the significance of p75ecd levels among different groups. Two groups were analyzed using the One-tailed t-test. More than two groups were analyzed using Dunn's Multiple Comparison Test in 1way ANOVA. The diagnostic accuracy of ALS was assessed using Receiver Operating Characteristic (ROC) curve analysis conducted with SPSS software, evaluating both sensitivity and specificity [7].

## 3 RESULTS

### 3.1 The Characteristics of the Participants Involved in the Study

A total of 60 ALS urine samples and 19 healthy control urine samples were included in the study. Table **1** provides the detailed features of the samples.

### 3.2 Urinary p75ecd Concentrations in ALS Patients *vs.* Healthy Control

**Fig. (1):**
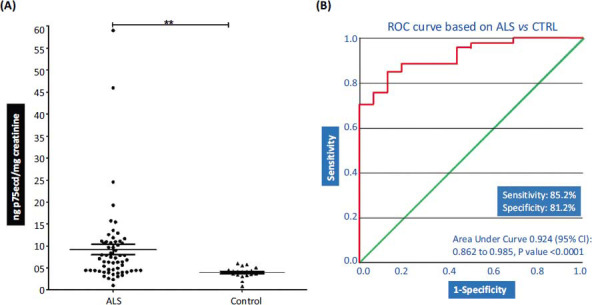
(**A**) Comparison of p75ecd in urine between ALS (n = 60) and control (n = 19) group by unpaired t-test (**P ≤ 0.01) and (**B**) Receiver operating characteristic curves for distinguishing ALS and control.

p75ecd concentration in the urine samples of ALS patients (9.229 ± 1.198 ng p75ecd/mg creatinine; n=60) is significantly higher as compared to control (3.979 ± 0.2891 ng p75ecd/mg creatinine; n=19) as shown in Fig. (**1A**). To evaluate the urinary p75ecd concentrations in ALS *vs.* Control, receiver operating characteristic (ROC) curves were generated. Urine p75ecd concentrations can distinguish ALS patients from Control, with area under the curve (AUC) of 0.924 (95% confidence limits of area 0.862-0.985; *P* value <0.0001 Fig. **1B**) with sensitivity 85.2% and specificity 81.2%.

### 3.3 Urinary p75ecd Concentrations in Male and Female ALS Patients *vs*. Healthy Control

**Fig. (2):**
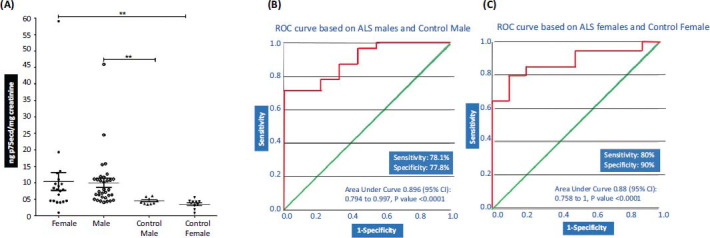
(**A**) Comparison of p75ecd in urine between ALS-male (n = 32); ALS-Female (n = 20) *vs.* Male Control (n = 9) and female control (n = 10) respectively by one way ANOVA (**P ≤ 0.01); (**B**) Receiver operating characteristic curves for distinguishing ALS males and male control; (**C**) Receiver operating characteristic curves for distinguishing ALS females and female control.

Further we divided the ALS patients sample and control samples on a gender basis and compared the concentration of p75ecd between ALS male and control Male and ALS female and control female. Interestingly, p75ecd levels were significantly higher in ALS males (9.961 ± 1.393; ng p75ecd/mg creatinine; n=32) as compared to healthy males (4.486 ± 0.3155 ng p75ecd/mg creatinine; N=9) and ALS females (10.45 ± 2.722 ng p75ecd/mg creatinine; n=20) as compared to healthy females (3.523 ± 0.4350 ng p75ecd/mg creatinine; n=10) (Fig. **2A**). To evaluate the urinary p75ecd concentrations in ALS males and females *vs.* Controls, ROC curves were generated. Urine p75ecd concentrations can distinguish ALS male patients from Male Control, with AUC of 0.896 (95% confidence limits of area 0.794-0.997; *P* value <0.0001 Fig. **2B**) with sensitivity 78.1% and specificity 77.8%%. Urine p75ecd concentrations can distinguish ALS female patients from Female Control, with an AUC of 0.88 (95% confidence limits of area 0.758-1; *P* value <0.001 Fig. **2C**) with sensitivity of 80% and specificity of 90%.

### 3.4 Urinary p75ecd Concentrations in Limb Onset (LO) ALS & Bulbar Onset (BO) ALS *vs.* Healthy Control

**Fig. (3):**
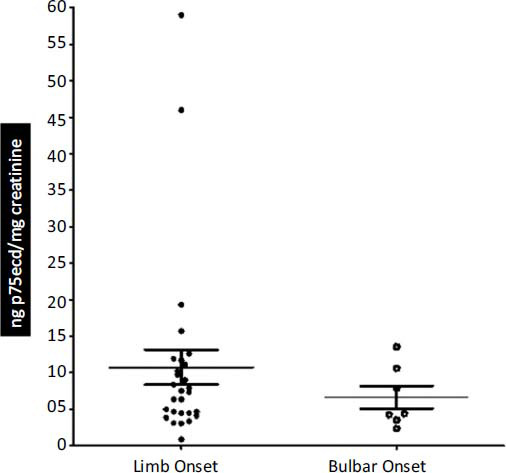
Comparison of p75ecd in urine between types of ALS (Limb Onset and Bulbar Onset).

Next, we divided ALS samples based on the type of ALS, BO ALS, and LO ALS. No significant difference was observed between the p75ecd levels of LO ALS (10.78 ± 2.385 ng p75ecd/mg creatinine; n = 28) and Bulbar Onset ALS (6.657 ± 1.573 ng p75ecd/mg creatinine; n = 7) (Fig. **3**).

### 3.5 Urinary p75ecd Concentrations in Various Age Groups of ALS *vs.* Healthy Control

**Fig. (4):**
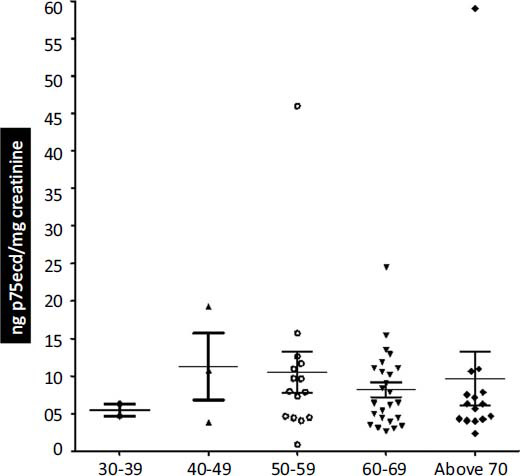
Comparison of p75ecd in urine among different age groups of ALS group by one way ANOVA.

Here we divided the ALS patients samples into various age groups (30-39; 40-49; 50-59; 60-69; above 70). The Mean ± SEM of all the age groups was, 5.498 ± 0.8490 ng p75ecd/mg creatinine; n = 2; 11.30 ± 4.469 ng p75ecd/mg creatinine; n = 3; 10.54 ± 2.723 ng p75ecd/mg creatinine; n = 15; 8.198 ± 1.012 ng p75ecd/mg creatinine; n = 25; 9.690 ± 3.578 ng p75ecd/mg creatinine; n = 15, respectively. No significant difference in the p75ecd levels was observed between the various age groups (Fig. **4**).

### 3.6 Urinary p75ecd Concentrations of ALS Patients with a Family History of Neurological Disorders *vs.* Healthy Control

**Fig. (5):**
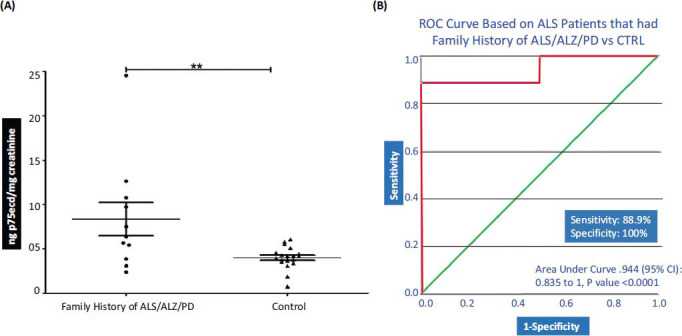
(**A**) Comparison of p75ecd in urine between Family history of ALS and control group by unpaired t-test (**P ≤ 0.01); (**B**) Receiver operating characteristic curves for distinguishing ALS patients that had family history of ALS/ALZ/PD and control.

Here the ALS patients' urine samples were included that had a family history (either parent) of neurological disorders and the levels of p75ecd were compared to Control samples that had no family history of neurological disorders. It was observed that the p75ecd levels were significantly high in the patients with a family history of ALS (8.352 ± 1.891 ng p75ecd/mg creatinine; n = 11) as compared to healthy controls (3.979 ± 0.2891 ng p75ecd/mg creatinine; n = 19) (Fig. **5A**). To evaluate the urinary p75ecd concentrations in ALS patients with a family history of neurological disorders *vs.* Control, ROC curves were generated. Urine p75ecd concentrations can distinguish ALS patients from Control, with AUC of 0.944 (95% confidence limits of area 0.835-1; *P* value <0.0001 Fig. **5B**) with sensitivity of 88.9% and specificity of 100%.

### 3.7 Urinary p75ecd Concentrations in ALS Patients that were Members of Armed Forces *vs.* Healthy Control

**Fig. (6):**
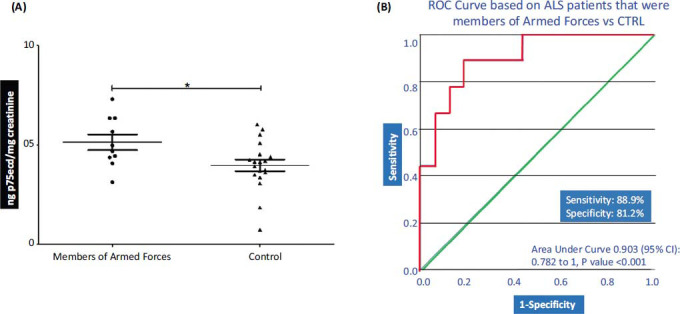
(**A**) Comparison of p75ecd in urine between ALS (Member of Armed Forces) and control group by unpaired t-test (*P ≤ 0.05); (**B**) Receiver operating characteristic curves for distinguishing ALS patients that were members of armed forces and control.

Here, only the samples that belong to the ALS patients that were members of armed forces were included and compared to the healthy control samples that never served in the armed forces. Our results show that the p75ecd levels were significantly high in the patients who were members of the armed forces (5.134 ± 0.4001 ng p75ecd/mg creatinine; n = 10) as compared to the healthy controls (3.979 ± 0.2891 ng p75ecd/mg creatinine; n = 19) (Fig. **6A**). To evaluate the urinary p75ecd concentrations in ALS patients who were members of armed forces *vs.* control, ROC curves were generated. Urine p75ecd concentrations can distinguish ALS patients from control, with AUC of 0.903 (95% confidence limits of area 0.782-1; *P* value <0.001 Fig. **6B**) with sensitivity 88.9% and specificity 81.2%.

### 3.8 Urinary p75ecd Concentrations in ALS Patients who Received Rilutek therapy *vs.* Non-receivers

**Fig. (7):**
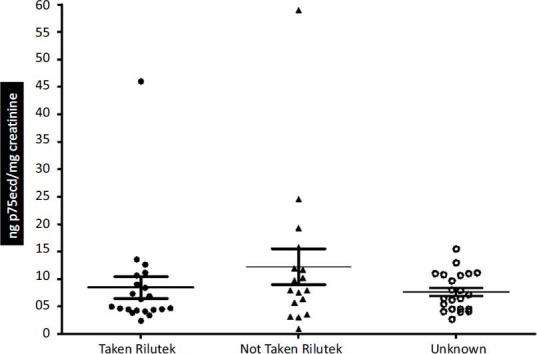
Comparison of p75ecd in urine among ALS patients with varied medication statuses (Taken Rilutek; Not taken Rilutek; Unknown medication status).

Here we divided the ALS patients based on medication (Rilutek) taken, not taken, and patients with unknown status. No significant difference in the levels of p75ecd was observed in the patients that had taken rilutek (8.443 ± 2.001 ng p75ecd/mg creatinine; n = 21), patients that had not taken rilutek (12.24 ± 3.278 ng p75ecd/mg creatinine; n = 17) and patients with unknown medical status (7.652 ± 0.7365 ng p75ecd/mg creatinine; n = 22) (Fig. **7**).

## 4 DISCUSSION

p75ecd is a useful candidate to indicate ALS progression [20]. Urinary biomarkers are of great interest because of the easy accessibility of the sample as most of the patients are readily willing to provide urine as compared to other biological fluids such as blood or CSF. Urine can be obtained non-invasively, whereas to provide blood or CSF, the patient had to undergo venipuncture or lumbar puncture. Since patient comfort and consent are of utmost consideration, especially in clinical trials, urine analysis appears feasible and more reasonable [6]. Our study utilized a cohort of 60 North American ALS patients and 19 healthy control urine samples. In this study, we observed a high concentration (approximately 2.4 times) of p75ecd in urine samples of ALS patients as compared to healthy controls (9.229 ± 1.198 *vs.* 3.979 ± 0.2891 ng p75ecd/mg creatinine). Levels of p75ecd in urine were normalized with urine creatinine [17] as this is a waste product of muscle metabolism and excreted in the urine at a relatively constant rate. Thus it is commonly utilized for the normalization of analyte concentrations in the urine [21]. The presence of urinary p75ecd is very low but detectable in healthy adult urine samples [22]. Studies conducted in Australian, and Chinese cohort [6, 7, 9] suggested elevated levels of p75ecd in the urine of ALS patients. Different cohort studies can be helpful in providing information to establish the relationship between the disease with geographical risk factors and the understanding of intricate interactions [23, 24].

Higher incidences of mortality due to ALS have been reported in Sweden, Finland, Norway, France, USA, New Zealand and Italy [25]. In the USA, the incidence rate of ALS ranges from 1.4 per 100,000 per year in California to 1.81 per 100,000 per year in Florida. The epidemiological analysis conducted, based on the ethnicity and race of samples in the USA suggests that the incidence rate of ALS was highest among European Americans (1.79 per 100,000 per year) in contrast to African Americans (0.80 per 100,000 per year) and Asian Americans (0.76 per 100,000 per year). Similarly, the incidence rate of ALS was higher in non-Hispanic (1.65 per 100,000 per year) residents of America in contrast to Hispanic (0.57 per 100,000 per year) American residents [26]. The annual cost associated with ALS in the US ranges between $212 million to $1.4 billion per year [27].

Thus, in this study, we also tried to establish the correlation between various factors with ALS. We categorized the ALS samples based on gender, type of ALS (LO/BO), age, family history, occupation, and medication status and evaluated the levels of p75ecd in these categories *versus* control. Although our results show a significantly elevated level of p75ecd in ALS patients as compared to healthy controls, there is no significant difference among ALS male and ALS female patients. Male sex has been considered a risk factor for ALS and previous studies show a high male-to-female ratio for ALS. However, as individuals age, the incidence of ALS tends to become more evenly distributed between men and women. In a study conducted by Batty *et al*., 25 risk indicators were studied in association with ALS, and the two most established risk factors observed were age and sex [28]. The reason for the decreasing male-to-female ratio may be due to equal almost exposure of men and women to ALS risk factors such as smoking, and occupational toxicants [29-31].

Next, we categorized the samples based on the type of ALS, limb onset (LO), and bulbar onset (BO) ALS. No significant difference was observed in the p75ecd levels in LO ALS *vs.* BO ALS which suggests that both LO and BO ALS exhibit the same levels of p75ecd levels. However, there was a limitation of low sample count for BO ALS samples (n=7) as compared to the samples for LO ALS (n=28). 70% of ALS cases exhibit limb onset that initiates with limb impairments, whereas 30% of ALS cases exhibit bulbar onset that is characterized by dysphagia, dysarthria, dysphonia & loss of facial muscle strength [32]. As the occurrence of LO ALS is more than BO ALS, it explains the low BO ALS samples among a total of 60 samples of ALS as the samples obtained from the biorepository were random. BO cases are associated with poor prognosis which in turn may be associated with overall muscle atrophy derived by malnutrition and this event may induce respiratory muscle weakness. The 5-year survival rate of BO ALS is 16% whereas for lower LO ALS is 21% and for upper LO ALS is 18% [33]. This information indicates that BO ALS is as significant as LO ALS, however, our results indicate the non-significance of BO ALS which may be due to the small sample size (28 for LO; 7 for BO) and could be ruled out in the future studies by conducting a similar study in a larger cohort. This suggests that p75ecd can serve as a universal marker for both LO ALS and BO ALS.

Furthermore, we grouped the samples based on age (30-39; 40-49; 50-59; 60-69; above 70). Our results indicate no significant difference in p75ecd levels among all the age groups. This indicates that p75ecd levels rise in ALS condition irrespective of age. Aging can play a pathogenic role in ALS as age-dependent dysregulation of RNA editing or RNA modifications may result in the accumulation of circular RNAs in the brain [34]. Before 40 years of age, ALS is rare but after that, it increases exponentially. The average age at ALS onset is 58-63 & 40-60 years for sporadic ALS & familial ALS respectively [25] whereas the average age at ALS diagnosis is 54-69 years [31]. In this study, among 60 random samples, the maximum samples belonged to the age group 50-69 (n=40) and only two samples were from the age group 30-39.

We also divided the ALS samples based on family history of motor neuron disease (MND) such as ALS, Alzheimer's, and Parkinson's. Our results indicate a significant difference in the levels of p75ecd between the patients that had a family history of MND *vs.* healthy controls that didn’t have any family history of MND. 10% of all ALS cases are familial fALS. fALS can be inherited either as an autosomal dominant or recessive trait. More than 150 disease-causing mutations have been identified and linked to ALS [35]. A study conducted in the Irish population suggests that from 1994 to 2016, an average of 1.9% (95% CI, 1.1-2.9) of confirmed ALS patients had a confirmed positive family history of frontotemporal dementia that increased by 0.2% each year [36]. The lifetime risk of ALS is estimated to be 1.1% in first-degree relatives (parents, siblings, and children) of individuals with ALS. This suggests a notable familial predisposition to the disease. Additionally, it appears that both environmental and genetic factors contribute equally to the development of ALS, highlighting the complex interplay between genetic susceptibility and environmental exposures in the pathogenesis of the disease [37]. ALS, PD, and ALZ share some common disturbances at the molecular level that include neuroinflammation, oxidative stress, mitochondrial dysfunction, metabolic dysregulation, inadequate protein quality control, *etc*. Along with shared molecular disturbances, these MNDs also share some genetic-level disturbances. Genome-wide association studies (GWAS) were used to identify the genetic overlap between these MNDs. The GWAS data suggested eleven common genetic loci between either two or all three of AD, PD, and ALS. These loci are related to lysosomal/autophagic dysfunction (GAK/TMEM175, GRN, KANSL1), neuroinflammation and immunity (TSPOAP1), oxidative stress (GPX3, KANSL1), and the DNA damage response (NEK1) [38]. This information supports our data that shows that a person with a family history of AD, PD, or ALS has a high risk of developing ALS.

Discussing risk factors, some specific risk factors for ALS include age, physical activity, alcohol consumption, smoking, diabetes mellitus, and military service. A study reveals that people with military service are 1.34 times more prone to develop ALS. The probable cause of this might be the occurrence of multiple exposures such as head trauma, organic solvents, viral infections, pesticides, formaldehyde, smoking, alcohol consumption [39-42]. The source of these exposures includes pollutants from unregulated industries, military vehicles, and aircraft emissions, emissions from open-air burn pits, particulate matter in desert environments, and toxicants present in military bases. Also, the soldiers receive cholinergic inhibitors as a prophylactic treatment against certain health risks. However, these prophylactic treatments have been linked to the potential risk of the development of neurodegenerative conditions such as ALS [40]. In our study, we categorized the ALS samples based on whether the patient had served in the armed forces and compared the levels of p75ecd among the patients who served in the armed forces *vs.* those healthy controls who never served in the armed forces. The results showed a significant increase in the levels of p75ecd in the patients who served in the armed forces as compared to control samples. Our results comply with the information that members of the armed forces are at higher risk of developing ALS.

Current treatment options for slowing ALS progression include three FDA-approved therapies, riluzole, edaravone, sodium phenylbutyrate, and taurursodiol. A fourth therapy, tofersen, was also approved under an accelerated approval [43]. In our current study, we divided the ALS patients sample based on the medication status (patients taken/not taken riluzole, and unknown medication status) and compared the levels of p75ecd among these subgroups. Our results indicate that all 3 groups had no significant difference in p75ecd levels. Riluzole has been identified to slow the progression of the disease and extend survival by a few months. The mechanism of action of riluzole is to reduce the release of the neurotransmitter glutamate which has damaging effects on motor neurons [44]. This indicates that Rilutek had no impact on the levels of p75ecd in ALS conditions.

This study is not without limitations. The number of samples are limited and are tested at only one time point thus, the progression status is missing along with the inclusion of other ALS mimic diseases that might be neurological or non-neurological. These shortcomings will be addressed in our future studies.

## CONCLUSION

This North American ALS cohort study suggests elevated levels of p75ecd in the urine of ALS patients. Our results are in accordance with previously published Australian and Chinese cohorts. This study further suggests that the development of an efficient system for the detection of elevated levels of p75ecd in the urine could serve as a useful modality for early ALS diagnosis, disease progression, and monitoring the effectiveness of therapeutic interventions. Our future objective will be to conduct this study in a larger cohort of patients, comparing the p75ecd levels in other neurological disorders as well as non-neurological disorders. Also, a combination of p75ecd with other biomarkers could further improve the efficacy, specificity, and sensitivity of this non-invasive test.

## Figures and Tables

**Table 1:** Demographic characteristics of ALS and control samples.

**Number of Samples**	**ALS: 60**	**Control: 19**
Gender	Male: 37 Female: 23	Male: 09 Female: 10
Type of ALS	Limb Onset: 28 Bulbar Onset: 7	Limb Onset: NA Bulbar Onset: NA
Age	30-39: 02 40-49: 03 50-59: 15 60-69: 25 70-79: 13 80-89: 02	20-29: 09 30-39: 06 40-49: 01 50-59: 03
Family History	Either Parent with ALS/ALZ/PD: 11	Either Parent with ALS/ALZ/PD: NA
Occupation	Member of Armed Forces: 10	Member of Armed Forces: NA
Medication	Taking Rilutek: 21 Not Taking Rilutek: 17 Unknown: 22	Taking Rilutek: NA Not Taking Rilutek: NA Unknown: NA

## Data Availability

All data generated or analyzed during this study are included in this published article.

## LIST OF ABBREVIATIONS

ALS: Amyotrophic Lateral Sclerosis
NfL: Neurofilament Light
pNfH: Phosphorylated Neurofilament Heavy
TNF: Tumor Necrosis Factor
NGF: Nerve Growth Factor
BDNF: Brain-derived Neurotrophic Factor
LO: Limb Onset
BO: Bulbar Onset
MND: Motor Neuron Disease
